# The Influence of Spiritual Leadership on Harmonious Passion: A Case Study of the Hotel Industry in China

**DOI:** 10.3389/fpsyg.2021.730634

**Published:** 2021-10-15

**Authors:** Yingda Wang, Yixing Jin, Lin Cheng, Ying Li

**Affiliations:** School of Tourism, Huangshan University, Huangshan, China

**Keywords:** calling, harmonious passion, spiritual leadership, self-determination, intrinsic motivation, psychological capital theory

## Abstract

The hotel manager has the responsibility to stimulate the passion of the staff. The vision, hope/faith, and altruistic love advocated by spiritual leaders can meet the independent psychological needs of employees, thus enhancing their harmonious passion. This study is based on self-determination theory, intrinsic motivation theory and psychological capital theory, and explores the relationship between spiritual leadership and employees’ harmonious passion. This study uses 260 employees of star hotels in Beijing, Shanghai, Hangzhou, Hefei, Huangshan, and other cities in China. Results show that spiritual leadership positively impacts employees’ harmonious passion, and calling plays an mediation role between spiritual leadership and employees’ harmonious passion. The results are helpful to clarify the formation mechanism of employees’ harmonious passion from the perspective of self-determination theory, intrinsic motivation theory and psychological capital theory and show that spiritual leadership can drive employees’ harmonious passion, especially when hotel vision and employee calling are consistent. Furthermore, the altruistic love of spiritual leaders for their followers also plays a key role in employee calling and promoting harmonious passion. Therefore, this study also emphasizes the importance of calling in improving the harmonious passion of employees. The theoretical and management implications that help to enhance the harmonious passion of employees are discussed in detail.

## Introduction

In recent years, one of the most critical challenges facing hospitality firms is an increasing decline in hospitable behaviors amidst intense commercialization and volatile competition within the sector (Usman et al., 2021a). The lack of passion of hotel employees has become an urgent problem to be solved. Lack of passion will reduce employees’ innovative behavior and have a negative effect on performance (Ho and Pollack, 2014). Individuals with low work passion are prone to low involvement in work and low job satisfaction, which is easy to produce burnout (Vallerand et al., 2010; Lavigne et al., 2012). Therefore, improving employees’ work passion has become an issue that hotel managers need to pay attention to.

Employees’ work passion is defined as a lasting, positive and meaningful state of health or happiness derived from their evaluation of work and organization (Zigarmi et al., 2009). Work passion is divided into harmonious work passion and forced work passion (Zigarmi et al., 2009). Harmonious passion emphasizes that individuals can enjoy their work, are willing to invest time and energy in their work, rather than be asked to do it, and do not need to obtain meaning and other oriented values or goals from their work (Dalla Rosa and Vianello, 2020).

Harmonious passion is positively correlated with job satisfaction, innovation, performance, employee well-being, work engagement, and employee health and negatively correlated with work-family conflict, workload, abnormal behavior, and violent behavior (Amabile and Mueller, 2008; Chen et al., 2009; Ho et al., 2011; Johan et al., 2011; Forest et al., 2012; Rip et al., 2012; Gousse-Lessard et al., 2013; Xu et al., 2013; Ho and Pollack, 2014; Lavigne et al., 2014; Astakhova and Porter, 2015; Zigarmi et al., 2015; Drnovsek et al., 2016; Sirén et al., 2016; St-Louis et al., 2016). Job satisfaction, innovation, and work commitment play an essential role in the sustainability and competitiveness of the organization (Douthwaite et al., 2009; Ashford and Hall, 2011; Marsden and Sonnino, 2012; Chen et al., 2014; Nwibere, 2014; Okeke and Mtyuda, 2017). Thus, more and more organizational managers and researchers begin to pay attention to the harmonious passion of employees. Thus, this study investigates the influence mechanism of the hotel staff’s harmonious passion, which is helpful to the hotel.

The existing research on the influencing factors of employees’ harmonious passion mainly has three aspects (Jiang et al., 2017). Firstly, the research has been carried out from the individual level. Harmonious passion is related to self-esteem, autonomous support climate, individual autonomy motivation, individual controllability perception, and goal pursuit tendency (Mageau et al., 2010; Lafrenière et al., 2011; Liu et al., 2011; Bélanger et al., 2015; Collewaert et al., 2016). Lafrenière et al. (2011) pointed out that individuals with higher explicit self-esteem implement more adaptive self-regulation strategies, which makes them more likely to produce harmonious passion. Mageau et al. (2010) proposed that individuals in independent support climate are easier to develop harmonious passion. Liu et al. (2011) believe that individual autonomy motivation contributes to the formation of harmonious passion. Secondly, the research has been carried out from the leadership level. Leadership behavior affects employees’ harmonious passion; spiritual leadership and authentic leadership can trigger the harmonious passion of employees’ active participation (Qin and Zhao, 2015; Afsar et al., 2016; Jia et al., 2018). Finally, the research has been carried out from the organizational level. Work autonomy support, team autonomy support and department autonomy support influence harmonious passion, which can positively predict harmonious passion (Liu et al., 2011; Fernet et al., 2014).

From the above literature on the influencing factors of harmonious passion, we find that there are few literatures on the relationship between leadership and harmonious passion, and the research on the impact of harmonious passion mainly focuses on Transformational Leadership (Jia et al., 2018; Li et al., 2020). However, the existing literature rarely discusses the influence mechanism of spiritual leadership on employees’ harmonious passion.

Psychological capital refers to the positive psychological state of an individual, including self-efficacy, optimism, hope, and resilience (Luthans and Youssef, 2004). As a way of leadership dedicated to meeting the spiritual needs of employees, spiritual leadership is bound to help employees maintain and develop positive psychological states or traits, so as to improve employees’ psychological capital (Yang et al., 2016). Positive social emotion and autonomy (psychological capital) can develop personal harmonious passion (Ho and Pollack, 2014; Ho et al., 2018). Spiritual leaders strengthen the internalization of the meaning of work, thus increasing the tendency or willingness of employees to devote time and energy to the work. The behavior pattern of spiritual leadership makes feeling the autonomous support climate easier for individuals, making them have autonomous motivation and leading to harmonious passion.

Spiritual leaders make their followers have a strong calling by building and conveying a clear and inspiring vision to their followers, strengthening their beliefs and caring for their followers. In contrast, those who have a strong sense of calling are likely to have harmonious passion and can actively devote themselves to their field of work (Dalla Rosa and Vianello, 2020). Therefore, this study will examine whether calling plays a mediation role between spiritual leadership and employees’ harmonious passion.

In addition, in recent years, the research on spiritual leadership is rising in the hotel industry. In order to improve the hospitality of employees, Usman et al. (2021a) proposed that managers should merge spiritual values with the traditional management techniques to explain and clarify the transcendental importance of their work and contributions. Previous hospitality literature suggests that workplace ostracism is also pervasive in the hospitality industry and represents a serious threat that undermines hospitality employees’ interpersonal relationships and the quality of social interactions (Ali et al., 2020a). spiritual leadership provided resources that enhanced employees’ social support at work and in doing so, reduced workplace ostracism (Ali et al., 2020a). Some key attributes of spiritual leadership are not only distinct from other related leadership styles but also imperative for hospitality firms, their employees, and customers. However, other types of leaders often ignore the importance of the spiritual values and needs of their followers (Usman et al., 2021a). Therefore, for hotel employees, the pursuit of spiritual value is very important.

From the above literature, it can be found that the research on spiritual leadership is very necessary for hotel management. Therefore, this study will take hotel employees as the research object to study the influence mechanism of spiritual leadership on employees’ harmonious passion, so as to enrich the literature of hotel management.

## Literature Review and Hypothetical Development

### Spiritual Leadership and Harmonious Passion

Although “spiritual leadership” has no unified definition in the current academic circles, in recent years, scholars from different cultural backgrounds tend to agree with Fry’s point of view: spiritual leaders can establish an organizational vision and organizational values on the basis of individual and team connections and combine their attitudes, values and behavior to make employees feel the significance of their work and the appreciation and understanding of the organization (Fry, 2003). Spiritual leadership has three intrinsic motivations, including altruistic love (reward), vision (performance), and hope/faith (effort) (Fry, 2003). Spiritual leaders emphasize the important role of employees’ high-level needs in giving full play to leadership effectiveness; leaders can motivate employees intrinsically by satisfying their sense of spiritual presence based on mission and membership to achieve results that are beneficial to individuals, groups (or teams) and organization (Fry, 2003).

Psychological capital refers to the positive psychological state of an individual, including self-efficacy, optimism, hope, and resilience (Luthans and Youssef, 2004). As a kind of leadership style dedicated to meeting the spiritual needs of employees, spiritual leadership is bound to help employees maintain and develop a positive psychological state or characteristics to improve their psychological capital (Yang et al., 2016). Spiritual leaders treat their followers with care, respect and confidence, which is likely to create a warm and caring environment (Chen and Li, 2013). This supportive organizational environment is conducive to developing employees’ psychological capital (Newman et al., 2014; Luthans and Youssef-Morgan, 2017).

Spiritual leadership helps employees realize the connection between work and the meaning of life, so that employees can have stronger hope/faith (Shi et al., 2018), and their own strong hope/faith can help them maintain a high degree of harmonious passion (Gao and Yan, 2018). The higher the level of psychological capital, the higher the passion (Cardon et al., 2009). Therefore, spiritual leadership has a positive impact on harmonious passion.

Organizational self-esteem is employees’ cognition and evaluation of their ability and value in the organization. Leadership behavior plays a vital role in the formation of employees’ organizational self-esteem. Employees will perceive whether they are essential or valuable in their organization through the information conveyed by their leaders or organizations (Pierce and Gardner, 2004). Altruistic love is one of the important contents of spiritual leaders. Spiritual leaders affirm their employees’ achievements in time and are tolerant when they are inadvertently out of date (Gu and Yu, 2020). Organizations and leaders’ concern, support and appreciation of employees can significantly improve employees’ level of organizational self-esteem (Yang et al., 2016). According to the intrinsic motivation theory, if employees think that they are important and valuable in their organization, they view their efforts to realize the organizational vision and be further motivated from within (Gu and Yu, 2020). Therefore, employees with high organizational self-esteem are likely to experience a high level of harmonious passion.

Furthermore, individuals with high explicit self-esteem use more adaptive self-regulation strategies because they are more responsive to situational cues (i.e., the degree of failure, the existence of alternatives, and the degree of progress), and self-regulation of multiple goals is also efficient (Di Paula and Campbell, 2002; Sedikides et al., 2007; Alicke and Sedikides, 2009). Therefore, these people are characterized by a high level of harmonious passion because they are likely to engage in relevant activities carefully while taking a correct view of other key areas of life. People with high external self-esteem will experience a high level of harmonious passion (Lafrenière et al., 2011).

According to self-determination theory, the satisfaction of the psychological needs of autonomy comes from two aspects: the psychological freedom perceived in completing the task and the self-satisfaction perceived in the process of listening to the leader’s advice (Van den Broeck et al., 2010). Spiritual leaders advocate the vision and faith of the coordination of organizational and personal interests, making employees feel more work significance, psychological freedom in the process of completing work tasks (Shi et al., 2018). Spiritual leaders care about and value employees, pay attention to upper and lower feedback in the process of interaction with employees and try their best to meet the needs of employees, which can implement the suggestions and tasks of spiritual leaders and improve employees’ satisfaction in the process of interaction (Shi et al., 2018). Thus, spiritual leadership can meet the basic psychological needs of employee autonomy, and promote the emergence of autonomous motivation (Shi et al., 2018). Being passionate about all activities is impossible, but the match between one’s interests, abilities and tasks is necessary to develop passion (Mageau et al., 2010). In an autonomous support climate, people are free to explore activities, demonstrate creativity, and eventually experience positive emotional outcomes (Mageau et al., 2010). Leaders who evoke positive emotions among their followers can stimulate a lasting sense of autonomous motivation for an activity, encouraging them to participate in out-of-role behavior (Dasborough and Ashkanasy, 2002). Positive social emotion and autonomy can develop personal harmonious passion (Ho and Pollack, 2014; Ho et al., 2018). To sum up, spiritual leaders can play an essential role in the generation of passion by building an autonomous support climate and supporting individual autonomous motivation.

Leadership is essentially an emotional process in which leaders show emotion and try to arouse the emotions of their members (Dasborough and Ashkanasy, 2002). Furthermore, through connections with others, society, self and transcendence, spiritual leaders help individuals become complete, seek meaningful work and become inspired to pursue higher goals and meaning (Hudson, 2014), stimulating passion for work. In addition, Ali et al. (2020b) found that spiritual leadership has a positive impact on harmony and security passion through empirical research. Anser et al. (2021b) pointed out that the direct relationship between spiritual leadership and OCBE depends on harmonious environmental passion.

Therefore, this study further infers that spiritual leadership may have a positive effect on harmonious passion. According to the above theory, the following hypothesis is put forward:

H1: Vision has a positive effect on harmonious passion.
H2: Hope/Faith has a positive effect on harmonious passion.
H3: Altruistic love has a positive effect on harmonious passion.

### Spiritual Leadership and Calling

There are many definitions of calling, as listed below:Calling is an individual’s ideal in career, and adults’ ideal of career is of great significance to their work (Richardson et al., 1978). Calling makes individual behavior affected by moral, social, and personal meaning satisfaction, which is the individual’s new career orientation (Wrzesniewski et al., 1997). Meaningful work can produce a calling experience, which can help individuals realize their self-worth and make society a better place (Hall and Chandler, 2005). Calling is a kind of work that gives individuals a sense of meaning in life and serves society (Duffy and Sedlacek, 2007). Calling is a transcendental calling experience that originates from the self and transcends self-experience. Its purpose is to realize a professional role that embodies or acquires a sense of purpose and regards the needs and social interests of others as the motivation for individuals to pursue the meaning and goals of life (Dik and Duffy, 2009). Calling is a process in which an individual pursues and achieves prosocial goals (Elangovan et al., 2010; Tian et al., 2012). Calling is a strong passion and power from the heart of an individual in response to a certain professional field (Dobrow and Tosti-Kharas, 2011). Chinese scholars have compared “calling” with the sense of mission under the Chinese cultural background and then put forward “calling” as the sense of mission (Zhang et al., 2012b). Calling is a continuous subjective psychological construct. Scholars have explored professional calling from the perspective of work motivation and subjective psychological perception and have attempted to carry out quantitative measurement and research. Career calling is considered specific to a career field and is a variable of continuous distribution from high to low (Dobrow and Tosti-Kharas, 2011). Career calling which can give individuals a sense of satisfaction in workplace is meaningful and beneficial to individuals, their family and society (Dobrow and Tosti-Kharas, 2011). The most representative is the study of Dobrow and Tosti-Kharas (2011), who believe that career calling is not an either or situation and everyone’s calling level is a continuous psychological construct between having and not having, which can be measured quantitatively (Pei and Zhao, 2015). The measurement object is a sense of satisfaction, a sense of achievement and the intensity associated with the meaning of an individual’s life between an individual and a specific professional field (Pei and Zhao, 2015).

Based on the previous studies, this research defined career calling as the subjective psychological perception of satisfaction and sense of life meaning, which obtained by individuals in their occupation.

Three intrinsic motivations in spiritual leadership, including altruistic love (reward), vision (performance), and hope/faith (effort) (Fry, 2003), increase people’s sense of spiritual existence, including calling, and membership (Rosa and Ancok, 2020). When leaders are spiritual, they can motivate employees to understand work well and directly (Mansor et al., 2013; Fry et al., 2017). Organizations need spiritual leaders because spiritual leaders will pay attention to and appreciate employees to make them feel that their work activities are valuable (calling) (Rosa and Ancok, 2020). Through the calling and membership among subordinates, spiritual leaders are always filled with a deep understanding of personal spirituality, the meaning and purpose of work, connection with the community and spiritual well-being (Duchon and Plowman, 2005).

By stimulating a sense of self-transcendence, organizational leaders may inspire a greater sense of calling and loyalty among their followers (Markow and Klenke, 2005). Organizations should focus on the vision and refine the vision to strengthen followers’ sense of transcendence (Markow and Klenke, 2005). The organization should also focus on values that allow followers to see their work as an opportunity to contribute their abilities and talents (Markow and Klenke, 2005). The variables that make up spiritual leadership (i.e., hope/faith, vision and altruistic love) constitute a high-level formative structure and positively impact on mental health (i.e., calling and membership) in the group (Fry et al., 2011). Spiritual leaders can create a meaningful vision for their followers to experience the meaning of life, feel a sense of mission and make changes (Wu and Lee, 2020). Many connections are found between spiritual leaders and employees’ career calling. Firstly, spiritual leaders focus on the spiritual needs of employees; that is, individuals pursue the demands of work and the meaning of life, whereas career calling points to the connection between work and the meaning of life. Secondly, spiritual leaders intend to increase individual internal drive, whereas employees with a sense of calling have a high level of internal drive (Shi et al., 2018).

To sum up, spiritual leadership has a significant positive impact on calling. These findings are consistent with the research of Fry et al. (2011) and Salehzadeh et al. (2015). Therefore, this study puts forward the following hypothesis:

H4: Vision has a positive effect on calling.
H5: Hope/faith has a positive effect on calling.
H6: Altruistic love has a positive effect on calling.

### The Mediation Role of Calling Between Spiritual Leadership and Harmonious Passion

The word “passion” comes from the Latin word “passio.” The word comes from Christianity and originally means “Jesus is willing to sacrifice himself to achieve a strong desire to save the world” (Vallerand et al., 2003). It implies suffering (Vallerand et al., 2003). At the end of the 20th century, scholars redefined passion from positive psychology, believing that “passion” is one of the important factors that make life more meaningful (Vallerand and Verner-Filion, 2013). Passion is pointed out to be a strong tendency or willingness of individuals to like (or even love), feel important (or extremely valuable) and require time and energy. Passion becomes a core feature of individual self-identity (Vallerand et al., 2003; Vallerand, 2012). In the organization field, the concept of passion for work originated from work commitment (Zhang et al., 2014). Work engagement is not enough to describe the relationship among emotion, cognition, will and behavior factors in social cognitive theory (Deci and Ryan, 2009). Thus, the researchers advocate that work passion should be used instead of work input and is a concept that goes beyond work input but can reflect the content of work in-put (Deci and Ryan, 2009). Based on self-determination theory (Deci and Ryan, 2000), scholars put forward the “dual model of passion,” which was gradually perfected and formed after several years of research (Vallerand et al., 2003).

According to the model, passion is “a strong tendency for people to participate in important activities they think they like. Because of this strong tendency, people invest their time and energy in this activity” (Vallerand et al., 2003). Individual passion can be divided into harmonious passion and obsessive passion. Harmonious passion comes from the internalization of autonomy, which leads to positive emotion and well-being (Birkeland and Buch, 2015). Obsessive passion stems from controlled internalization, which may produce a sense of maladjustment and lead to negative emotions (Birkeland and Buch, 2015). Subsequently, scholars apply the dual model of passion in organizational behavior and divide employees’ work passion into harmonious work passion and obsessive work passion. Employees’ work passion is defined as a lasting, positive and meaningful state of health or happiness derived from their evaluation of work and organization (Zigarmi et al., 2009). At present, the vast majority of scholars in academic circles have adopted the dual model of work passion. Among them, harmonious passion refers to the strong motivational tendency of the individual to an activity. Still, the individual with harmonious passion has the right of choice and can control himself to participate in the activity freely. Therefore, the harmonious passion of employees plays a positive role in the development of organizations and individuals (Vallerand et al., 2003).

Harmonious passion is different from conceptually similar concepts, such as work involvement, work engagement, calling, and workaholism (Lajom et al., 2018). Calling and harmonious passion refer to activities that people love and often participate in and define their identity. However, individuals with high-level calling feel driven to the calling domain by something greater or beyond themselves (transcendental calling). Participation in the calling domain gives them a sense of purpose (purpose). Their activities in the calling domain are beneficial or good for others (prosocial orientation) (Lajom et al., 2018). The definition of work passion does not include these calling elements. Specifically, harmonious passion is different from calling because individuals can enjoy their work. They find harmonious passion important and devote time and energy to it without realizing that they have been asked or called to do the job and not derive meaning and other-oriented values or goals from their work (Dalla Rosa and Vianello, 2020).

However, the core of harmonious passion is a structure with a strong relationship with calling in theory. Individuals are likely to be enthusiastic about things of social importance (Robertson and Barling, 2013). Those who see their profession as calling are likely to be enthusiastic about their work activities (Bunderson and Thompson, 2009; Peterson et al., 2009; Dobrow and Tosti-Kharas, 2011). Those who feel a high level of calling tend to show commitment to work (Hirschi, and Andreas, 2012). The more people connect at work and reveal themselves, the more they invest and are more passionate about their tasks (Lips-Wiersma, 2003). Career calling is often associated with people’s enthusiasm and passion for work-related activities, which are considered fascinating, critical and worth the time and effort (Vallerand et al., 2003; Berg et al., 2010). Furthermore, the empirical study found that calling is positively related to harmonious passion but has nothing to do with obsessive passion (Lajom et al., 2018).

To sum up, the vision advocated by spiritual leaders achieves a high degree of coordination between individual and organizational goals, making employees understand that efficient and high-quality work is beneficial to them and leaders, organizations and society as a whole and realize the significance of work. Spiritual leaders care for their employees, provide as much help as possible and create an organizational environment conducive to their growth, making them feel the attention from the organization and realize the significance of their work. Spiritual leaders can motivate employees with beliefs, realize that the goal will eventually be achieved and further understand the importance of work. Spiritual leadership enhances employees’ connection with the meaning of work and makes employees feel a calling. Employees with a high calling are likely to be passionate in their work and actively devote themselves to their field of work. Thus, this study puts forward the following hypothesis:

H7: Calling has a positive effect on harmonious passion.
H8: Calling has a mediating effect between vision and harmonious passion.
H9: Calling has a mediating effect between hope/faith and harmonious passion.
H10: Calling has a mediating effect between altruistic love and harmonious passion.

**FIGURE 1:**
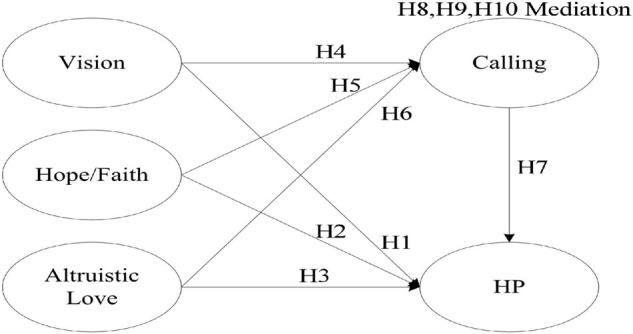
Proposed conceptual framework.

This study proposes the development of a structural model of vision, hope/faith, calling, altruistic love and harmonious passion. Figure 1 is the proposed conceptual framework of this study.

## Materials and Methods

### Samples and Procedures

We randomly selected 25 luxury hotels from Beijing, Shanghai, Hangzhou, Hefei, Huangshan, and other cities and randomly chose 280 frontline employees to issue paper questionnaires forthwith. We gained their support by communicating with the hotel leadership. We also informed the hotel staff that the survey was completely anonymous and optional. We encouraged them to participate and pointed out that the study would contribute to the hotel’s development. To help reduce the common method variance effect, we distributed two questionnaires (Podsakoff et al., 2003). A 2-month lag is considered appropriate for reducing common method variance, which has been used in several studies (Anser et al., 2021a; Usman et al., 2021a,b). The first round of the survey measured demographic information, spiritual leadership, and calling. In the second round, with the help of the hotel manager, we found employees to participate in the first round of the survey and asked them to complete the harmonious work passion scale. We distributed 280 questionnaires in total. Given that 20 responses had missing data, we excluded them from further data analyses and hypotheses testing. Thus, the actual effective questionnaires were 260. Of the 260 respondents, over 97% were between 18 and 45 years old. A total of 91% of the respondents had a lower than college degree. In total, 45.77% of the employees have worked for 1–3 years. A total of 11.92% of the employees have worked for 4–6 years. A total of 14.62% of the employees have worked for 7–10 years. A total of 20% of the employees have worked for over 10 years. Among the hotels surveyed, most (59.23%) were four-star hotels, 22.31% were three-star hotels, and 18.46% were five-star hotels (Table 1).

**TABLE 1:** Participants’ profile (*n* = 260).

**Socio-demographic indicators *N* (%)**	***N***	**%**	**SD**
**Age (*n* = 260)**			0.803
18–25 years old	103	39.62%	
26–35 years old	112	43.08%	
36–45 years old	39	15.00%	
46–55 years old	6	2.31%	
**Education level (*n* = 260)**			0.988
Technical school education or below	96	36.92%	
high school education	72	27.69%	
College degree	70	26.92%	
Bachelor’s degree or above	22	8.46%	
**Working year (*n* = 260)**			1.312
6 months–1 year	20	7.69%	
1–3 years	119	45.77%	
4–6 years	31	11.92%	
7–10 years	38	14.62%	
Over 10 years	52	20.00%	
**Gender (*n* = 260)**			0.488
Female	159	61.15%	
male	101	38.85%	
**Department (*n* = 260)**			1.606
Food and beverage	104	40.00%	
Front office	26	10.00%	
Housekeeping	54	20.77%	
Executive office	14	5.38%	
Other departments	62	23.85%	
**Hotel type (*n* = 260)**			0.826
Three stars	58	22.31%	
Four stars	154	59.23%	
Five stars	48	18.46%	

### Measurement

All the surveys were translated from English to Chinese and then translated/back using a translation program (Brislin, 1970). Five hotel experts (i.e., two hospitality management professors, one general manager, and two HR managers) were invited to adjust the wording of the scale test items on the basis of the basic situation of the hotel and Chinese language habits while guaranteeing that the original meaning remains unchanged to ensure that the respondents can clearly understand the project’s content. All measurements use the Likert scale, ranging from 1 “strong disagreement” to 5 “strong agreement.”

A 17-item spiritual leadership scale developed by Fry et al. (2005) was used. The scale contains three dimensions: vision, hope/faith, and altruistic love (Fry et al., 2005). Sample items for spiritual leadership include “my hotel’s vision is clear and compelling to me” and “the leaders in my hotel are honest and without false pride.” The Cronbach’s alpha values of the three scales were 0.93, 0.93, and 0.91. The overall reliability of the spiritual leadership scale is 0.94.

The calling scale is a 12-item scale developed by Dobrow and Tosti-Kharas (2011). The reliability and validity of the scale were strictly tested. The internal consistency reliability of the occupational call scale was between 0.87 and 0.95. Zhang et al. (2012a) and Pei and Zhao (2015) used this scale in their study and achieved good results. Finally, the calling scale in this study was developed on the basis of Pei and Zhao’s (2015) revised scale. Sample items for calling include “the work I do is meaningful to me” and “the work I do makes a difference in people’s lives.” The Cronbach’s alpha of the calling scale was 0.91.

Harmonious passion adopted the work passion scale developed by Vallerand et al. (2003). The scale was divided into two parts and has 14 items, of which seven items tested harmonious passion and the other seven items tested obsessive passion (Vallerand et al., 2003). In this study, seven items of harmonious passion were measured. Sample items for harmonious passion include “this activity allows me to live a variety of experiences” and “this activity reflects the qualities I like about myself.” The Cronbach’s alpha was 0.91.

## Analysis and Results

### Confirmatory Factor Analysis Testing and Descriptive Statistics

To validate the model, we estimated five different models. They are five-, four-, three-, two-, and single-factor models. The five-factor model includes vision, hope/faith, altruistic love, calling, and harmonic passage. In the four-factor model, we combined calling and harmonious passion. In the three-factor model, we combined vision, hope/faith, and altruistic love. In the two-factor model, we merged the calling and harmonious passion into one factor and merge vision, hope/faith, and altruistic love into one factor, simultaneously. Finally, in the single-factor model, we combined calling, harmony, passion, vision, hope/faith, and altruistic love into one factor. We conducted a CFA using Amos 23.0. Table 2 shows that the fitting index of the five-factor model (basic model) showed an acceptable degree of fit [*x*^2^ = 926.36, df = 584, *x*^2^/df = 1.59 (<3), *C* = 0.05 (<0.08), CFI = 0.95 (>0.90), IFI = 0.95 (>0.90), GFI = 0.82 (>0.80)]. Hair et al. (2006) recommended that the GFI and CFI are best if they are above 0.90 and are acceptable if they are above 0.80. Bollen (1989) suggested that if the IFI is higher than 0.90, it is best. Browne and Cudeck (1993) proposed that when the RMSEA is less than 0.05, the model fitting is good and basically acceptable when between 0.05 and 0.08. In the three-factor model, two- and single-factor models, the RMSEA value was higher than 0.08 and was not within the recommended value range. In the four-factor model, the GFI was lower than 0.8 and was not within the recommended value range. Thus, none of the four models was accepted. Through the above comparison, we conclude that the fitting index of the five-factor model is better than that of other models.

**TABLE 2:** Results of confirmatory factor analysis.

Model	Factors	GFI	IFI	TLI	CFI	RMSEA
Five-factor model	C, HP, V, HF, AL	0.82	0.95	0.95	0.95	0.05
Four-factor model	C+HP, V, HF, AL	0.72	0.90	0.89	0.90	0.07
Three-factor model	C, HP, V+HF+AL	0.62	0.81	0.82	0.80	0.09
Two-factor model	C+HP, V+HF+AL	0.57	0.76	0.74	0.76	0.11
Single-factor model	C+HP+V+HF+AL	0.55	0.73	0.71	0.73	0.11

*C, calling; HP, harmonious passion; V, vision; HF, hope/faith; AL, altruistic love.*

We calculated composite reliability (CR) and average variance extracted (AVE) for the latent constructs (Liu et al., 2020). The CR coefficients should be greater than 0.7, and the AVE values should be greater than 0.5 (Hair et al., 2006). We analyzed the validity scale by focusing on content validity, convergent validity, and discriminant validity (Liu et al., 2020). As shown in Table 3, the loadings of all items to their respective factors were statistically significant, ranging from 0.64 to 0.89 (all > 0.40). The loadings of all items in the final measurement model exceeded the minimum cut-off point of 0.40 (Hair et al., 2014a,b), achieving internal consistency. The CR of each construct was between 0.90 and 0.95, and all values were over 0.70 (Hair et al., 2006). In addition, Cronbach’s α exceeded the recommended minimum reliability standard 0.70 (Nunnally, 1978). In terms of convergence validity, all CR values were higher than the minimum cut-off point of 0.70, and all AVE values met the lowest standard of 0.50 (Fornell and Larcker, 1981). Thus, the reliability, convergent validity and discriminant validity of the measurement model were satisfactory and provided sufficient evidence.

**TABLE 3:** Result of measurement model.

Construct		Factor loading	α	CR	AVE	α	CR	AVE
**Calling**	**Calling**		0.95	0.95	0.60	0.95	0.95	0.60
	C1	0.78						
	C2	0.75						
	C3	0.77						
	C4	0.81						
	C5	0.74						
	C6	0.79						
	C7	0.81						
	C8	0.83						
	C9	0.86						
	C10	0.72						
	C11	0.67						
	C12	0.81						
**Harmonious passion**	**HP**		0.90	0.91	0.58	0.90	0.91	0.58
	HP1	0.75						
	HP2	0.64						
	HP3	0.76						
	HP4	0.81						
	HP5	0.69						
	HP6	0.87						
	HP7	0.80						
**Spiritual leaders**	**Vision**		0.93	0.93	0.72			
	V1	0.77						
	V2	0.84						
	V3	0.87						
	V4	0.85						
	V5	0.86						
	**HF**		0.93	0.93	0.72			
	HF1	0.84						
	HF2	0.89						
	HF3	0.78				0.94	0.94	0.58
	HF4	0.87						
	HF5	0.84						
	**AL**		0.91	0.91	0.60			
	AL1	0.73						
	AL2	0.69						
	AL3	0.84						
	AL4	0.81						
	AL5	0.68						
	AL6	0.84						
	AL7	0.82						

*P < 0.001, HP, harmonious passion; HF, hope/faith; AL, altruistic love.*

As shown in Table 4, the results showed a significant correlation among vision, hope/faith, altruistic love, calling, and harmonious passion. These significant correlations provide a necessary basis for hypothesis testing. Discriminant validity is established when the AVE value for each construct is greater than the squared correlation coefficients for the corresponding inter-construct correlations (Kline, 2005). As shown in Table 4, the AVE value of each construct was over 0.50, and the square root of AVE was greater than the correlation. Therefore, convergence validity and discriminant validity were satisfactory.

**TABLE 4:** Results of descriptive statistics and correlations.

	Mean	SD	AVE	C	HP	V	HF	AL
C	3.70	0.73	0.60	(0.78)				
HP	3.84	0.68	0.58	0.69^**^	(0.76)			
V	3.82	0.73	0.72	0.63^**^	0.65^**^	(0.85)		
HF	3.95	0.74	0.72	0.65^**^	0.62^**^	0.51^**^	(0.85)	
AL	3.69	0.76	0.60	0.72^**^	0.71^**^	0.62^**^	0.57^**^	(0.77)

**P < 0.01. C, calling; HP, harmonious passion; V, vision; HF, hope/faith; AL, altruisticlove. The square root of AVE is on the diagonal. Off diagonals are Pearson correlation of constructs.

### Hypothesis Test

**FIGURE 2:**
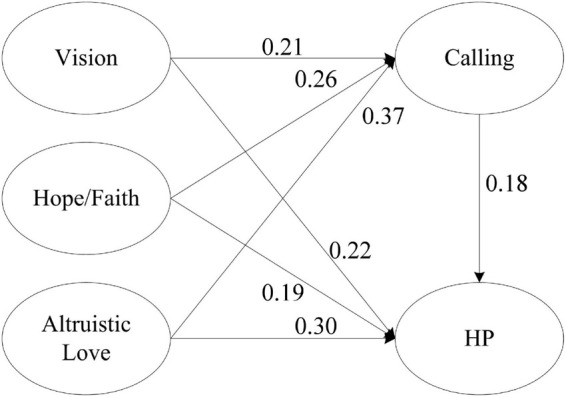
Final structural model with different path results.

Figure 2 shows the final structural model with direct path results. Vision positively affects harmonious passion. Hope/faith positively affects harmonious passion. Altruistic love positively affects harmonious passion. Vision positively affects calling. Hope/faith positively affects calling. Altruistic love positively affects calling. Calling positively affects harmonious passion.

The value and significance level of each path coefficient indicated that the structural path was positive and significant, hence all direct positive relationships are supported (Table 5). As shown in Table 6, the total effects of vision, hope/faith, and altruistic love on harmonious passion are positive and significant (β = 0.26, *P* < 0.001, β = 0.23, *P* < 0.001, β = 0.36, *P* < 0.001), thus supporting H1, H2, and H3. Table 5 shows the direct effects of vision, hope/faith, and altruistic love on harmonious passion are significant (β = 0.22, *P* < 0.001, β = 0.19, *P* < 0.001, β = 0.30, *P* < 0.001), which further support hypothesis. After increasing the mediating variables of calling, the effect size of vision on harmonious passion decreased from the total effect of 0.26 (*P* < 0.001) to the direct effect of 0.22 (*P* < 0.001), thus supporting H8; the effect size of hope/faith on harmonious passion decreased from the total effect of 0.23 (*P* < 0.001) to the direct effect of 0.19 (*P* < 0.001), thus supporting H9; the effect size of altruistic love on harmonious passion decreased from the total effect of 0.36 (*P* < 0.001) to the direct effect of 0.30 (*P* < 0.001), thus supporting H10.

**TABLE 5:** Path results for the final structural model (hypothesis testing).

Hypothesis/path	Coefficient	*t*-value	Result
H1: Vision->HP	0.22	3.51^***^	Supported
H2: Hope/Faith->HP	0.19	3.51^***^	Supported
H3: Altruistic Love->HP	0.30	4.44^***^	Supported
H4: Vision->Calling	0.21	3.48^***^	Supported
H5: Hope/Faith->Calling	0.26	5.19^***^	Supported
H6: Altruistic Love->Calling	0.37	5.97^***^	Supported
H7: Calling->HP	0.18	2.30^*^	Supported

*P < 0.05, ***P < 0.001.HP, harmonious passion.

**TABLE 6:** Direct and mediation effects.

Paths	Point estimate	Product of coefficients		Bootstrapping				Two-tailed significance
				Percentile 95% CI		Bias-corrected 95% CI		
		SE	Z	Lower	Upper	Lower	Upper	
Vision→Calling→HP (indirect effects)	0.04	0.02	2.00	0.001	0.092	0.004	0.099	<0.050
Vision→HP (direct effects)	0.22	0.07	3.03	0.083	0.373	0.094	0.390	<0.001
Total effects	0.26	0.08	3.45	0.123	0.416	0.130	0.424	<0.001
H/F→Calling→HP (indirect effects)	0.05	0.02	2.24	0.001	0.108	0.001	0.109	<0.050
H/F→HP (direct effects)	0.19	0.06	3.32	0.077	0.297	0.080	0.302	<0.001
Total effects	0.23	0.05	4.30	0.125	0.343	0.127	0.344	<0.001
AL→Calling→HP (indirect effects)	0.07	0.03	2.17	0.127	0.463	0.132	0.466	<0.050
AL→HP (direct effects)	0.30	0.09	3.48	0.001	0.158	0.002	0.160	<0.01
Total effects	0.36	0.07	5.23	0.225	0.496	0.224	0.494	<0.001

*Standardized estimating of 2000 bootstrap sample.*

Vision had a significant positive predictive effect on calling (β = 0.21, *P* < 0.001), thus supporting H4. Hope/faith had a significant positive predictive effect on calling (β = 0.26, *P* < 0.001), thus supporting H5. Altruistic love had a significant positive predictive effect on calling (β = 0.37, *P* < 0.001), thus supporting H6. Calling had a significant positive predictive effect on harmonious passion (β = 0.18, *P* < 0.05), thus supporting H7.

The non-parametric percentile Bootstrap method of deviation correction was used to test the mediation effect, the sampling was repeated 2,000 times, and the 95% confidence interval was calculated. The results showed that vision, hope/faith, and altruistic love indirectly affected harmonious passion through calling (β = 0.04, *P* < 0.001; β = 0.05, *P* < 0.001; β = 0.07, *P* < 0.001, respectively), the path coefficient of each variable and the effect value of the mediating path are shown in Figure 2 and Table 6.

The 95% confidence interval of the mediating effect from vision to harmonious passion was (0.004, 0.099), and the range did not include 0, and *Z* > 1.96; the 95% confidence interval of the mediating effect from hope/faith to harmonious passion was (0.001, 0.109), and the range did not include 0, and *Z* > 1.96; the 95% confidence interval of the mediating effect from altruistic love to harmonious passion was (0.132, 0.466), and the range did not include 0, and *Z* > 1.96, indicating that all the mediating effects of the model was significant, thus supporting H8, H9, and H10.

The research findings support all hypotheses explicitly.

## Discussion

The results support all hypotheses. This research examines the effect of three dimensions (vision, hope/faith, and altruistic love) of spirit leaders in hotels, and put forward a conceptual framework to improve the harmonious passion of hotel employees. The study also investigated the mediating role of calling in the relationship between spiritual leadership and harmonious passion. Ten hypotheses are tested, and the following statistically significant conclusions are drawn. These causal relationships are as follows: vision, hope/faith and altruistic love positively affect calling and harmonious passion, and calling has a positive direct effect on harmonious passion. Calling also acts as a mediation between vision, hope/faith, altruistic love, and harmonious passion. The potential variables’ overall internal consistency and structural validity show that the scale has good reliability and validity. Firstly, vision has a significant effect on calling and harmonious passion. Specifically, for every increase of one vision, the number of calling increases by 0.21, and that of harmonious passion increases by 0.22. Hope/faith has a significant effect on calling and harmonious passion. Specifically, for each increase of 1 hope/faith, the number of calling increases by 0.26, and that of harmonious passion increases by 0.19. Finally, altruistic love had a significant effect on calling and harmonious passion. Specifically, for each increase of one altruistic love, the calling increased by 0.37, and harmonious passion increased by 0.30.

We analyzed the effect of calling and harmonious passion from three dimensions of spiritual leadership. Firstly, the vision advocated by spiritual leaders makes individual goals highly coordinated with organizational goals, which makes employees realize the significance of work and that their work is beneficial to themselves, the organization and society. Motivating employees to find the meaning of career can change their attitude and behavior (Aksu, 2005; Poulston, 2008); thus, employees who realize the meaning of work will be full of passion in their work. At the same time, spiritual leaders promote employees’ connection with the meaning of work by advocating vision to make them feel a sense of calling. The promotion of calling promotes employees to be full of passion in their work. Secondly, spiritual leaders can strengthen employees’ beliefs and help them see the goals, improve their morale and realize that the goals will eventually be achieved, stimulating the harmonious passion of employees. At the same time, spiritual leaders stimulate employees’ sense of calling by helping them to establish hope/faith to enhance their harmonious passion. Finally, spiritual leaders care for their employees, provide as much help as possible and create an organizational environment conducive to their growth, making them feel the attention from the organization and the significance of their work and become more passionate in their work. Thus, spiritual leadership enhances employees’ connection with the meaning of work and makes employees feel a sense of calling. Employees with a high sense of calling are likely to be passionate at work and actively devote themselves to their field of work, which is consistent with the results of previous studies (Vatou and Gkorezis, 2018).

After calling is added to the model, the factor of spiritual leadership effectively promotes the improvement of employees’ harmonious passion, which provides preliminary evidence for the positive relationship between spiritual leadership and calling. The results also show that, in hotel management, spiritual leaders will affirm hotel employees’ achievements in time, which improves employees’ organizational self-esteem and makes employees feel that their work is important and valuable. In other words, spiritual leadership improves employees’ calling, which further makes employees feel that they are important and valuable in their organization, so employees will experience a higher level of harmonious passion. Thus, the theory of internal motivation explains the possibility that spiritual leaders can improve the harmonious passion of employees (Gu and Yu, 2020).

Furthermore, the positive correlation between calling and harmonious passion revealed in this study is consistent with previous research conclusions (Wu and Lee, 2020), which emphasize the positive role of calling in improving the meaning of work and harmonious passion. For hotel managers, improving employees’ sense of calling can improve employees’ prosociality and enhance their perceived professional significance and internal motivation (Tong, 2014). It can make employees more passionate and emit more powerful energy (Gao, 2013). The results show that calling affects harmonious passion and mediates the relationship between spiritual leadership and harmonious passion. This attempt is the first to use calling as a mediation between the two. The spiritual leadership practice of hotel managers [encouraging, accepting, and supporting diversified expressions of employees’ spiritual values (Leigh, 1997)] on the basis of the evaluation intensity of relevant spiritual values, conduct selection, orientation, socialization, performance evaluation, and skills training for new employees (Fry and Altman, 2013; Anderson et al., 2017) and establishing a compelling vision supported by organizational values of hope and altruistic love to stimulate the subjective well-being of participants in the organization (Fry, 2003) is promoted to improve employees’ sense of calling. Their psychological capital, happiness and sense of work meaning and the level of harmonious passion of employees can be improved. Our findings provide important information about leadership development practices for organizations (such as hotels) and individuals.

## Conclusion

The research results make a significant theoretical contribution to the leadership research and supplement the effect of spiritual leadership on harmonious passion. This study empirically studies that spiritual leadership is an important determinant of calling, consistent with previous studies (Fry, 2003). This study also contributes to the literature on leadership research by revealing the mediation role of calling. The research on the outcome variables of existing spiritual leaders extends to corporate profits, sales growth, sustainability, financial performance and social responsibility, employees’ ethical well-being, spiritual well-being and life satisfaction (Zhang and Ling, 2011). However, research on the antecedent variables that affect these result variables is not deep enough. Research on the effect of spiritual leadership on harmonious passion has been limited, and scholars have not mentioned the research on calling as a mediation between spiritual leadership and harmonious passion. The present study reveals the mediation role of calling and emphasizes the importance of “meaning of work.” Therefore, this study also contributes to the model construction of stimulating harmonious passion: spiritual leadership and calling directly impact harmonious passion. Calling can also mediate the relationship between spiritual leadership and harmonious passion. This study proposes a new conceptual framework to understand the determinants and results of harmonious passion. The theoretical framework and research results can be used as the basis for future research.

In view of the serious consequences of hotel employees’ lack of passion on hotel competition and development, and more and more people call for studying the role of different positive leadership styles in improving employees’ harmonious work passion, this study is timely and relevant. This study is an important step to extend the spiritual leadership theory to hotel management and empirically show the positive relationship between spiritual leadership and employees’ harmonious passion. Our research results provide a theoretical basis for adopting a spiritual leadership model in the hotel and provide an effective solution for hotel managers on how to improve the harmonious passion of their employees. Harmonious passion has a significant positive impact on job satisfaction, innovation, performance, employee well-being, work commitment and employee health (Ho et al., 2011; Forest et al., 2012; Lavigne et al., 2014; St-Louis et al., 2016). Therefore, the management is responsible for enhancing the employees’ harmonious passion and encouraging the hotel to adopt the spiritual leadership model to stimulate the harmonious passion of the staff, which improves the development of the hotel.

By conveying the hotel’s vision to the hotel staff, hotel managers can describe a clear and desirable blueprint for the development of the hotel and show sincere care to the hotel staff. Doing so stimulates their strong sense of professional calling and makes them feel that the construction and development of the hotel will be different because of them. Thus, their professional identity is promoted, and their harmonious passion is improved. Specifically, to give employees a better experience of the hotel vision, managers can create an organizational atmosphere with a sense of challenge, making employees more innovative and challenging. Furthermore, to make employees feel the hotel’s concern, they can be encouraged to express their ideas and care about their humanized needs. Finally, to strengthen their beliefs, hotel managers should lead by example to win respect and trust of their staff and help employees build the belief that their goals will be achieved.

Hotel managers can evaluate career calling in the recruitment, selection and staffing of their hotel staff. In the process of hotel staff recruitment, various testing tools should be used to measure the career calling level of employees, and those employees with a high sense of career calling should be selected. The fit between hotel employees’ personal sense of professional mission and corporate vision should also be evaluated to encourage the selected employees have a more harmonious passion in their future work. The vision of the hotel in the construction of the hotel culture should be emphasized, and the vision of the hotel should be combined with the personal career calling of the employees to make the career calling of the hotel staff fit with the culture of the hotel and improve the employees’ harmonious work passion. Hotel managers should strengthen the work meaning education in the training and the hotel staff’s true love for the profession by setting an example.

Hotel staff should be given more independence and encouraged to actively participate in the formulation of relevant management rules and methods conducive to the realization of their professional mission and significance. By providing flexible conditions, highlighting the importance of work, empowering them, and rewarding their initiatives, managers can make their work more enjoyable and inspire them to internalize work activities into their identity (Usman et al., 2021a), which can better motivate them. Salary and bonus are no longer the only major motivators. Employees can be retained by motivating them from the perspective of meeting the pursuit and mission of life. Thus, the hotel should also pay attention to the realization of social value, note the establishment of social image and provide hotel employees with a vision of social value to attract employees with career calling to join and promote the development of the hotel (Zhou and Li, 2018).

## Limitations and Directions for Future Research

The first restriction is related to the sampling samples of this study. The data are from some provinces and cities in China but are mainly from some tourist cities. Thus, the evidence from other areas is limited, which may lead to sampling bias. Considering the differences in the degree of development and culture of hotels in different provinces and cities in China, the leadership styles adopted by hotels in different regions have different effects on their employees. Although the participants show differences in age, education, working years and gender, future studies should consider collecting data on various types of hotels with a larger sample size in different income regions of China and even in other countries. Furthermore, this study collected data on spiritual leadership, calling and harmonious passion through the hotel staff. Given the potential bias that may be caused by self-assessment, future research should examine the impact of spiritual leadership on harmonious passion through a team or organization to address this limitation.

For the second restriction, although spiritual leadership and calling have a positive impact and mediation effect on employees’ harmonious passion, the cross-sectional study design of this study limits the generality of the prediction of the causality. Therefore, future research should also consider more variables, such as job autonomy, workplace exclusion and self-esteem, and take more methods to study, such as experimental and longitudinal research methods, to address these limitations.

## Data Availability Statement

The raw data supporting the conclusions of this article will be made available by the authors, without undue reservation.

## Ethics Statement

Ethical review and approval was not required for the study on human participants in accordance with the Local Legislation and Institutional Requirements. The patients/participants provided their written informed consent to participate in this study.

## Author Contributions

YW and YJ conceived the study. YW, YJ, LC, and YL wrote the manuscript. All authors designed the study, collected and analyzed the data, read and approved the manuscript, and agreed to be accountable for all aspects of the work.

## Conflict of Interest

The authors declare that the research was conducted in the absence of any commercial or financial relationships that could be construed as a potential conflict of interest.

## Publisher’s Note

All claims expressed in this article are solely those of the authors and do not necessarily represent those of their affiliated organizations, or those of the publisher, the editors and the reviewers. Any product that may be evaluated in this article, or claim that may be made by its manufacturer, is not guaranteed or endorsed by the publisher.
